#  Multistrategy Self-Organizing Map Learning for Classification Problems

**DOI:** 10.1155/2011/121787

**Published:** 2011-08-16

**Authors:** S. Hasan, S. M. Shamsuddin

**Affiliations:** Soft Computing Research Group, Faculty of Computer Science and Information System, Universiti Teknologi Malaysia, Skudai, 81300 Johor, Malaysia

## Abstract

Multistrategy Learning of Self-Organizing Map (SOM) and Particle Swarm Optimization (PSO) is commonly implemented in clustering domain due to its capabilities in handling complex data characteristics. However, some of these multistrategy learning architectures have weaknesses such as slow convergence time always being trapped in the local minima. This paper proposes multistrategy learning of SOM lattice structure with Particle Swarm Optimisation which is called ESOMPSO for solving various classification problems. The enhancement of SOM lattice structure is implemented by introducing a new hexagon formulation for better mapping quality in data classification and labeling. The weights of the enhanced SOM are optimised using PSO to obtain better output quality. The proposed method has been tested on various standard datasets with substantial comparisons with existing SOM network and various distance measurement. The results show that our proposed method yields a promising result with better average accuracy and quantisation errors compared to the other methods as well as convincing significant test.

## 1. Introduction

In classification process; normally, large classes of objects are separated into smaller classes. This approach can be very complicated due to the challenge in identifying the criteria especially for procedures involving complex data structures. In this scenario; practically, the Machine Learning (ML) techniques will be used and introduced by many researchers as alternative solutions to solve the above problems. Among the ML methods and tools, Artificial Neural Network (ANN), Fuzzy Set, Genetic Algorithm (GA), Swarm Intelligence (SI), and rough set are commonly used by researchers. 

However, the most popular ML method widely used by the practitioners is ANN [1]. Various applications of ANN which have been implemented in many practical problems such as meteorological forecasting, image processing, and agriculture are discussed in [2–4]. In ANN model, simple neurons are connected together to form series of connected network. While a neural network does not have to be adaptive, its advantages arise with proper algorithms to update the weights of the connections to produce a desired output.

ANN and evolutionary computation methodologies have each been proven effective in solving certain classes of problems. For example, neural networks are very efficient at mapping input to output vectors and evolutionary algorithms are very useful at optimization. ANN weaknesses could be solved either by enhancing the structures of ANN itself or by hybridizing it with evolutionary optimisation [5, 6]. Evolutionary computation is based on population of optimisation techniques such as evolutionary Algorithm (EA) and Swarm Intelligence (SI). One of the techniques used in EA is Genetic Algorithm (GA), inspired by biological evolution such as inheritance, mutation, selection, and crossover. On the other hand, SI methods such as Particle Swarm Optimisation (PSO) and Ant Colony Optimisation (ACO) are motivated by flock of birds, swarm of bees, ant colony, and school of fish.

The searching implementation with evolutionary method such as ANN learning may overcome the gradient-based handicaps. However, the convergence is in general much slower, since these are general purpose methods. Kennedy and Eberhart [7] proposed a very simple nonlinear optimisation technique called PSO which requires little computational costs. The authors argued that PSO could train Feedforward Neural Network (FNN) with a performance similar to the backpropagation (BP) method, for the XOR and Iris benchmarks. Since then, several researchers have adopted PSO for FNN learning. However, most of the studies focus on the hybridisation of PSO and FNN. Few studies have been conducted on the hybridisation of PSO with Self-Organizing Map (SOM) to solve complex problems.

Early studies have shown that the multistrategy learning of PSO-SOM approach was first introduced by Shi and Eberhart [8] with modified particle swarm optimizer. Subsequently, Xiao et al. [9, 10] used hybrid SOM-PSO approach to produce better clustering of gene datasets. The authors used SOM learning and PSO to optimise the weights of SOM. However, the merit for combination of SOM-PSO without conscience factor was poor than SOM alone. This is because this factor is valuable as a competitive learning technique, but it reduces the number of epochs necessary to produce a robust solution. In 2006, O'neill and Brabazon [11] adopted PSO as unsupervised SOM algorithm. The authors suggested using different distance metric in calculating the distance between input vectors and each member of the swarm to produce competitive result for data classification. However, in this study, types of SOM lattice structure were not considered.

Moreover, Chandramouli [12] used SOM and PSO for image classifier, and the author stated that SOM was dominant in image classification problems. However, the problem emerged in generating image classes which provided concise visualisation of the image dataset. Therefore, the author used dual layer of SOM structure and PSO to optimise the weights of SOM neurons. In addition, Forkan and Shamsuddin [13] introduced a new method for surface reconstruction based on hybridization of SOM and PSO. The authors used growing grid structure in the Kohonen network to learn the sample data through mapping grid and PSO to probe the optimum fitting points on the surface. In this study, the proposed Kohonen network was a 3D rectangular map and being enhanced using growing grid method. However, this study did not focus on the lattice structure of the Kohonen network.

Sharma and Omlin [14] utilized a U-matrix of SOM to determine cluster boundaries using PSO algorithm. The authors compared the results with other clustering techniques such as k-means and hierarchical clustering. However, this study did not focus on the structure of SOM architecture. Recently, Özçift et al. [15] proposed PSO in the optimisation of SOM algorithm to reduce the training time without loss of quality in clustering. The author stated that the size of lattice is related to the clustering quality of SOM. This optimisation technique has successfully reduced the numbers of nodes that finds the best-matching unit (BMU) for a particular input. Having a larger grid size in SOM will invite higher training time. Furthermore, the larger the lattice size, the more nodes should be considered for BMU calculation, thus causes higher operating cost for the algorithm. 

Since 1993, Extension of SOM network topologies such as self-organization network has been implemented in many applications. Fritzke [16] introduced Growing Cell Structures (GCS) for unsupervised learning in data visualisation, vector quantization and clustering, while supervised GCS is suitable for data classification with Radial Basis Function (RBF). In 1995, Fritzke extended the growing neural gas to dynamic SOM known as Growing SOM (GSOM). Hsu et al. [17] stated that GSOM can provide balance performance in topology preservation, data visualization, and computational speed. Consequently, Chan et al. [18] used GSOM to improve binning process and later Forkan and Shamsuddin [13] for intelligent surface reconstruction.

Hybridization of SOM and evolutionary method was proposed by Créput et al. [19] to address the vehicle routing problem with time windows. In this study, the experimental result shows that the proposed method improves SOM-based neural network application. In addition, Khu et al. [20] implemented the combination of multiobjective GA and SOM to incorporate multiple observations for distributed hydrologic model calibration. SOM clustering has reduced the number of objective functions while multiobjective GA was implemented to give better solution in optimization problems. Furthermore, Eisuke [21] investigated GA performance by combining GA with SOM to improve search performance by real-coded GA (RCGA). The result shows that SOM-GA gives better solution in computation times rather than RCGA.

**Figure 1:**
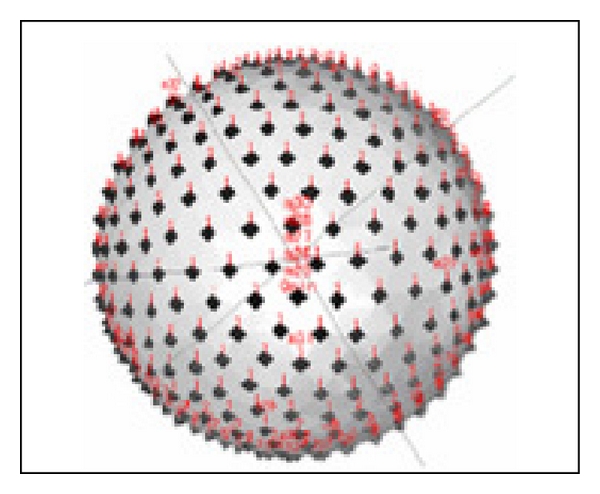
Spherical SOM [24].

**Figure 2:**
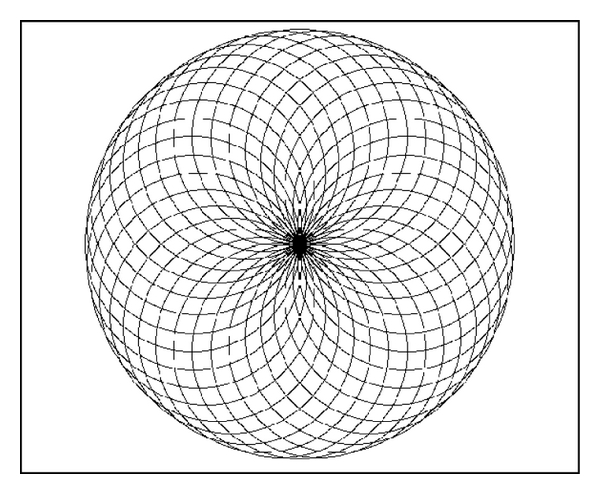
Torus SOM. http://altnett.ning.com/profiles/blogs/the-sphere.

The quality of the Kohonen map is determined by its lattice structure. This is because the weights for each neuron in the neighborhood will be updated by these lattice structures. There are many types of SOM lattice structures: circle lattice structure, rectangular, hexagonal, spherical (Figure 1), and torus (Figure 2). Many studies have been done on comparing the lattice structure of SOM, for instance, between traditional SOM and Spherical [22, 23]. 

 Spherical and Torus SOM representing the plane lattice give a better view of the input data as well as provide closer links to edge nodes. They make the 2D visualisation of multivariate data possible using SOM's code vectors as data source [24]. Spherical and Torus SOM structures focus on topological grid mapping structures rather than improvement on lattice structure. This is due to the border effect issues that were highlighted in previous studies by Ritter [25] Marzouki and Yamakawa [26]. Furthermore, the usefulness of the Spherical SOM for clustering and visualization is discussed in [27, 28].

According to Middleton et al. [29], hexagonal lattice structure is effective for image processing since the structure can uniform the image pixel. Park et al. [30] has used the hexagonal lattice to provide better visualization. Hexagonal lattice was preferred because it does not favor horizontal or vertical directions [31]. The number of nodes was determined as $5\times \sqrt{\text{number}\;\text{of}\;\text{samples}}$ [32]. Basically, the two largest eigenvalues of the training data were calculated, and the ratio between the side lengths of the map grid was set to the ratio between the two maximum eigenvalues. The actual side lengths were set so that the product was close to the determined number of map units. 

Astel et al. [33] has also implemented the hexagonal neighborhood lattice and compared the SOM classification approach with cluster and Principal Component Analysis (PCA) for large environmental dataset. The results obtained allowed detecting natural clusters of monitoring locations with similar water quality type and identifying important discriminant variables responsible for the clustering. SOM clustering allows simultaneous observation of both spatial and temporal changes in water quality.

Wu and Takatsuka [34] used fast spherical SOM for geodesic data structure. The proposed method was used to remove border effect in SOM, but the limitation was slower in computation times. Furthermore, Kihato et al. [24] implemented spherical and torus SOM for analysis, visualization, and prediction of the syndrome trends. The proposed method has been implemented by physicians to monitor patients' current health trends and status.

Due to limitations of the previous studies focusing on the improvement of SOM lattice structure, this study enhanced SOM lattice structure with improved hexagonal lattice area. In SOM competitive learning process, wider lattice are needed for searching the winning nodes as well as for weights adjustment. This allows SOM to get a good set of weights for improving the quality of data classification and labeling. Particle Swarm Optimisation (PSO) is developed to optimize SOMs' training weights accordingly. The hybridisation of SOM-PSO architecture, so-called Enhanced SOM with Particle Swarm Optimisation (ESOMPSO) is proposed with improvement on the lattice structure for better classification. The performance of the proposed ESOMPSO is validated based on the classification accuracy and quantization errors (QE). The error deviations between the proposed methods are computed to further illustrate the efficiency of these approaches accordingly.

## 2. The Proposed Method

In this study, we proposed multistrategy learning with the Enhancement of SOM with PSO (ESOMPSO) and improved formulation of hexagonal lattice structure. Unlike conventional hexagonal lattice (as given in (1)), a neighbourhood of the proposed formulation is given with the influence of *N*(*j*, *t*) instead of the neighbourhood width, *N*(*j*). Since *D*(*t*) is a threshold value, it will decrease gradually as training progresses. For this neighbourhood function, the distance is determined by considering the distance of each dimension. The dimension with the maximum value is chosen as distance node from BMU, *d*(*j*). *N*(*j*) corresponds to a hexagonal lattice around *n*_win_ having neighbourhood width as below:

(1) $$R=6\times \frac{1}{2}\times r\times \sqrt{\left(r^{2}\right)-\left(\frac{1}{4}r^{2}\right)},$$

where *R* is the standard hexagonal lattice and

(2) $$N\left(j,t\right)=\{\begin{array}{c} 1,d\left(j\right)\leq D\left(t\right)\\ 0,d\left(j\right)>D\left(t\right). \end{array}$$

The weights of all neurons within this hexagon are updated with *N*(*j*) = 1, while the others remaining unchanged. As the training progresses, this neighborhood gets smaller, resulting in the neurons that are very close to the winner and will get updated accordingly. The training stops when there is no more neuron in the neighborhood. Usually, the neighborhood function, *N*(*j*, *t*), is chosen as an L-dimensional Gaussian function as given below:

(3) $$N\left(j,t\right)=\exp\;\frac{-d{\left(j\right)}^{2}}{2\sigma {\left(t\right)}^{2}}.$$

The proposed SOM algorithm for the above process is shown below.

For each input vector *V*, do the following.

**Figure 3:**
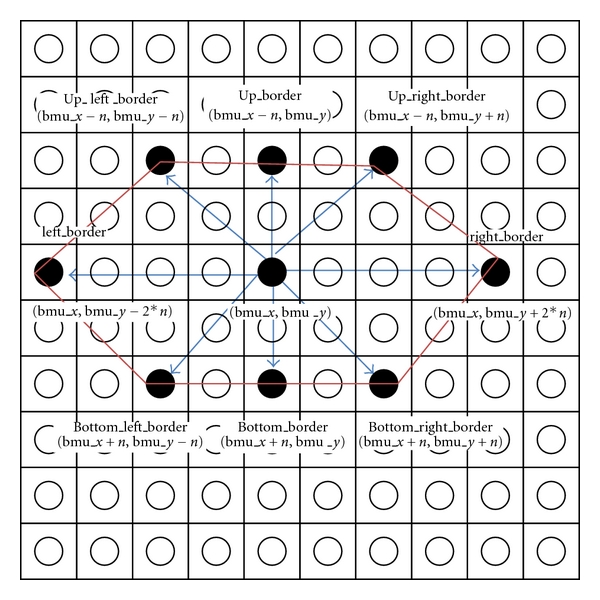
The proposed lattice structure for enhanced SOM (ESOM).

- Initialisation—set initial synaptic weights to small random values, say in a interval [0,1], and assign a small positive value to the learning rate parameter.
- Competition—for each output node *j*, calculate the value *D*(*V* − *W*_*j*_) of the scoring function. For instance, Euclidean distance measurement is denoted as
  (4a) $$D\left(V-W_{j}\right)=\sqrt{\overset{i=n}{\underset{i=0}{\sum }}{\left(V_{i}-W_{ij}\right)}^{2}},$$
  for the Manhattan distance, the equation is given as
  (4b) $$D\left(V-W_{j}\right)=\overset{i=n}{\underset{i=0}{\sum }}(V_{i}-W_{ij}),$$
  for the Chebyshev distance, the equation is given as
  (4c) $$D\left(V-W_{j}\right)=\underset{i}{\max\;}\left|V_{i}-W_{ij}\right|.$$
  Find the winning node *J* that minimizes *D*(*V* − *W*_*j*_) overall output nodes.
- Cooperation—identify all output nodes *j* within the neighborhood of *J* defined by the neighborhood size *R*. For these nodes, do the following for all input records. Reduce the radius with exponential decay function:
  (5) $$\sigma \left(t\right)=\sigma_{0}\exp\;\left(-\frac{1}{\lambda }\right),\;t=1,2,3,\ldots ,$$
  where *σ*_0_ is the initial radius, *λ* is the maximum iteration, *t* is the current iteration; and formulation of improved hexagonal lattice is given as
  (6) $$R_{\text{new}}={\left(2r+1\right)}^{2}+2r^{2},$$
  where *R*_new_ is the enhanced/improved hexagonal lattice, *r* is the neighborhood radius.
- Adaptation—adjust the weights:
  (7) W(t+1)=W(t)+Θ(t)L(t)(V(t)−W(t)),
  where *L* is the learning rate, Θ is the influence a node's distance from the BMU,
  (8) $$L\left(t\right)=L_{0}\exp\;\left(-\frac{t}{\lambda }\right),\;t=1,2,3,\ldots ,$$
  where *L*_0_ is the initial learning rate,
  (9) $$\Theta \left(t\right)=\exp\;\left(-\frac{{\mathrm{dist}\;}^{2}}{2\sigma^{2}\left(t\right)}\right),\;t=1,2,3\ldots ,$$
  and dist is the distance of a node from BMU, *σ* is the width of neighborhood.
- Iteration—adjust the learning rate and neighborhood size, as needed until no changes occur in the feature map. Repeat step (ii) and stop when the termination criteria are met. The improved hexagonal lattice area consists of six important points: right_border (*x*, *y*), left_border (*x*, *y*), up_right_border (*x*, *y*), up_left_border (*x*, *y*), bottom_right_border (*x*, *y*), bottom_left_border (*x*, *y*) (see Algorithm  1).  Figure 3 illustrates the formulation of improved hexagonal lattice area. Detail explanation of the proposed method is discussed in next paragraph.

Subsequently, the weights of ESOMPSO learning are optimised by PSO. Particle Swarm Optimisation (PSO) is one of the Swarm Intelligence (SI) techniques that are inspired by social behavior of bird flocking and fish schooling. The pioneers of the PSO algorithm are Kennedy, Eberhart, and Shi in 1995 [35]. PSO is a global optimisation, population-based evolutionary algorithm for dealing with problems in which the best solution can be presented as a point or surface in an *n-*dimensional space. Hypothesis are plotted in this space and seeded with an initial velocity, as well as a communication between the particles. In this study, the hybridisation approach of ESOMPSO is based on the Kohonen structure to improve the quality of data classification and labeling. An improved hexagonal lattice area is introduced for SOM learning enhancement; and PSO is integrated into this proposed SOM to evolve the weights for the learning prior to the weights adjustments. This is because PSO can find the best reduced search space for a particular input and support the algorithm to take more nodes into consideration while determining search space and not to be trapped by the same node continuously [15]. The algorithm for integrating ESOMPSO is shown below. At this stage, the enhanced SOM will be implemented for the classification purpose to obtain the weights and later will be optimised using PSO.

1. The rectangular topology and hexagonal lattice structure of the SOM is initialized with feature vectors *m*_*i*_, where *i* = 1,2,…, *K* randomly, where *K* is the length of the feature vector.
2. Input feature vector *x* is presented to the network and the winner node *J*, that is closest to the input pattern, *x is chosen *using the equation:
  (10) $$J=\text{ar}{\text{g}}_{i}\min\;\left\{||x-m_{j}||\right\}.$$
3. Initialise the population array of particle representing random solutions for *d* dimensional problem space.
4. For each particle, the distance function is evaluated,
  (11) $$D_{ij}=\overset{k}{\underset{l=1}{\sum }}\left|x_{ij}-x_{jl}\right|.$$
5. The personal best *p*best is updated by the following condition:
  (12) $$\begin{array}{c} \text{if}\;\left(f\left(p\text{bes}{\text{t}}_{i}\right)>\text{curren}{\text{t}}_{i}\right),\;\text{then}\;p\text{bes}{\text{t}}_{i}=\text{curren}{\text{t}}_{i}. \end{array}$$
6. The global best *g*best is updated with the following condition:
  (13) $$\begin{array}{c} \text{if}\;\left(f\left(g\text{bes}{\text{t}}_{d}\right)=f\left(\text{curren}{\text{t}}_{d}\right)\right),\;\text{then}\;g\text{bes}{\text{t}}_{d}=\text{curren}{\text{t}}_{d}. \end{array}$$
7. Update the velocity *V*_*id*_ using
  (14) $$V_{id}=WxV_{id}+C1\left(G_{\text{best},d}-X_{id}\right)+C2\left(P_{\text{best},i}-X_{id}\right),$$
  where *C*1 > 0  and  *C*2 > 0 constants are called the *cognitive *and *social *parameters, and *W* > 0 is a constant called the *inertia *parameter.
8. Update the position *X*_*id*_ using
  (15) $$X_{id}=X_{id}+V_{id},$$
  where *X*_*id*_ is the new position *X* and *V*_*id*_ is the new velocity *V*.
9. Repeat steps 2 to 9 until all input patterns are exhausted in the training.

## 3. Experimental Setup

To investigate the effectiveness of PSO in evolving the weights from SOM, the proposed method has been performed in the testing and validation process. In the testing phase, data is presented to the network with target nodes for each input sets. The reference attributes or classifier computed during training process is used to classify input data set. The algorithm identifies the winning node that will be used for determining the output of the network. Then, the output of the network is compared to the expected result to decide the ability of the network for classification phase. This classification stage will classify test data into correct predefined classes obtained during training process. A number of data is presented to the network, and the percentage of correct classified data is calculated. The percentage of the correctness is measured to obtain the accuracy and the learning ability of the network. The result is validated and compared using several performance measurements: quantisation error (QE) and classification accuracy. Later, the error differences between the proposed methods are computed for further validations.

The performance measurement of the proposed methods is based on quantisation error (QE) and classification accuracy (CA). QE is measured after SOM's training, and CA is the analysis for testing. The efficiency of the proposed methods is validated accordingly; if QE values are smaller and the classification accuracy is higher, then the results are promising. QE is used for measuring the quality of SOM map. QE of an input vector is defined by the difference between the input vector and the closest codebook vectors. QE describes how accurately the neurons respond to the given dataset. For example, if the reference vector of the BMU calculated for a given testing vector *x*_*i*_ is exactly similar as *x*_*i*_, the error in precision is 0.0. The equation is given as follows

Quantization Error:

(16) $$E_{q}=\frac{1}{N}\overset{N}{\underset{k-1}{\sum }}||x_{k}\left(t\right)-w_{mk}\left(t\right)||,$$

where *w*_*mk*_ is the best unit of weight on times *t*. 

While the classification accuracy indicates how well the classes are separated on the map, the classification accuracy of new samples measures the networks generalisation for better quality of SOM's mapping. 

Classification accuracy,

(17) $$P\left(\%\right)=\frac{n}{N}\times 100,$$

where *n* is the number of classified pattern, *N* is the total number of testing data.

The goal of the conducted experiments is to investigate the performance of the proposed methods. The comparisons are done on ESOMPSO, SOM with PSO (SOMPSO), and enhanced SOM (ESOM). The results are validated in terms of classification accuracy and quantisation error (QE) on standard universal machine learning datasets: Iris, XOR, Cancer, Glass, and Pendigits. From the conducted experiments, it shows that the proposed methods, ESOMPSO and SOMPSO, give better accuracy despites higher convergence time. As PSO and improved lattice structure are being implemented, the convergence time is increasing. This scenario is due to the PSO process in searching for the *g*best of BMU as well as wider coverage for updating nodes with the improved lattice structure.

Self-Organizing Maps (SOM) has two layers: input and output layers. The basic SOM architecture consists of a lattice that acts as an output layer with its input nodes fully connected. In this study, the network architecture is designed based on the selected real world classification problems.  Table 1 provides the specification for each dataset.

The input layer is comprised of input pattern with different nodes that is randomly chosen from training data set. Input patterns are presented to all output nodes (neurons) in the network simultaneously. The number of input node determines the number of data required to be fed into the network, while the numbers of nodes in the Kohonen layer represent the maximum number of possible classes.  Table 2 shows the class information for Iris, XOR, Glass, and Pendigits training datasets. 

The training starts once the dataset has been initialised and input patterns have been selected. The learning phase of the SOM algorithm repeatedly presents numerous patterns to the network. The learning rule of the classifier allows these training cases to organize in a two-dimensional feature map. Patterns which resemble each other are mapped onto a specific cluster. During the training phase, the class for randomly selected input node is determined. This is done by labeling the output node that is more similar (best-matching unit) to the input node compared to other nodes in the Kohonen mapping structure. The outputs from the training are the resulting map that contains the winning neurons and its associated weight vectors. Subsequently, these weight vectors are optimised by PSO. The quality of the classification accuracy is calculated to investigate the behavior of the network in the training data. 

In the testing phase, for any input patterns, if the *m*th neuron is the winner, it belongs to the *m*th clusters. In this case, we were able to test the capacity of the network to correctly classify new independent test set to a reasonable class. An independent test set is a set similar to the input set but not part of the training set. The testing set can be seen as a representative of the general case. There is no weight updating in the recalling phase. A series of datasets obtained that was not used in learning phases, but was previously interpreted, was presented to the network. For each case, the response of the network (the label of the associated neuron) was compared to the expected result, and the percentage of correct responses was computed. This simulation results obtained from standard SOM and Enhanced SOM classifiers were used for further analysis. 

It is often reported in the literature that the success of the Self-Organizing Maps (SOM) formation is critically dependent on the initial weights and the selection of main parameters of the algorithm, namely, the learning rate parameter and the neighborhood set [36, 37]. They usually have to be counteracted by trial and error method, hence time consuming to retrain the procedures. Due to the time constraints, all the parameter values were fixed and constantly used throughout all the experiments. According to [38], the number of map units is usually in the range of 100 to 600. Deboeck and Kohonen [39] recommend using ten times the dimension of the input patterns as the number of neurons, and this was adopted in these experiments.

There is no guideline in suggesting good learning rates to any given learning problem. In standard SOM, too large and too small learning rates can lead to poor network performance [40]. Neighborhood function and the number of neurons determine the granularity of the resulting mapping. Larger neighborhood was used in the beginning of training and then gradually decreases to a suitable final radius. The larger the area for neighborhoods functions with high values, the more rigid and flexible the map will be. In these experiments, the initial radius size is set to half of the size of the lattice. A more recent version of the feature map adapts the Gaussian function to describe the neighborhood and the learning rate. The Gaussian function is supposed to describe a more natural mapping so as to help the algorithm converge in a more stable manner.

The accuracy of the map also depends on the number of iterations of the SOM algorithm. A rule of thumb states, for good statistical accuracy, number of iterations should be at least 500 times the number of neurons. According to [36], the total learning time is always 100 to 10000. If the time taken is longer, the clustering result becomes inaccurate. A more serious problem is that the topology preserving mapping is not guaranteed even if a huge number of iterations were used. Here, the SOM classifiers were evaluated by measuring the performance of clustering result based on the classification accuracy and the computation time [41]. To meet the requirement of SOM's quality measurement, the quantisation error was calculated, which is defined as the average distance between every input vector and its BMU. The experiments on ESOMPSO were carried out for each selected dataset (Table 3).

## 4. Experimental Results and Analysis

The experiments were conducted with various datasets and distance measurements: Euclidean, Manhattan, and Chebyshev distance. The comparisons were conducted between standard SOM and standard SOM with improved hexagonal structure, so-called ESOM. Standard SOM was trained using standard hexagonal lattice, while ESOM with improved hexagonal lattice. The choice of distance measure influences the accuracy, efficiency, and generalisation ability of the results. From Table 4, ESOM with Euclidean distance gives promising accuracy of 86.9876%, followed by the Chebyshev distance 84.2561% and the Manhattan distance 80.4462%. The least quantisation error is 0.0108 for Glass dataset. It shows that the improved lattice structure of ESOM yields significant impact on the accuracy of the classifications.

Similar experiments were conducted for standard SOM with PSO, so-called SOMPSO and ESOMPSO with Euclidean, Manhattan's, and Chebyshev's distance measurements. SOMPSO was trained using standard hexagonal lattice, while ESOMPSO was trained with improved hexagonal lattice. The results were compared in terms of classification accuracy, quantisation error, and convergence error. As illustrated in Table 5, ESOMPSO provides the least error distance for searching the particles nearest to the input vector. It shows that improved lattice structure of ESOM yields significant impact on the accuracy of the classifications despite slower convergence time. This is due to the usage of larger lattice structure in ESOMPSO. By having larger grid size, higher training time will be generated. Furthermore, the larger the lattice size is, the more nodes for BMU calculation are to be considered. However, in this study the focus is on the performance of the proposed method based on higher accuracy and lower QE.

**Figure 4:**
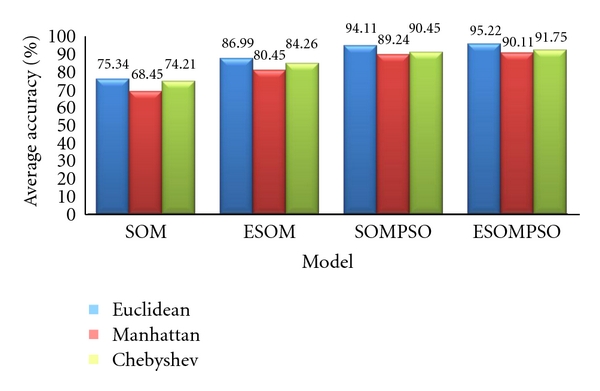
Accuracy SOM, ESOM, SOMPSO, and ESOMPSO.

**Figure 5:**
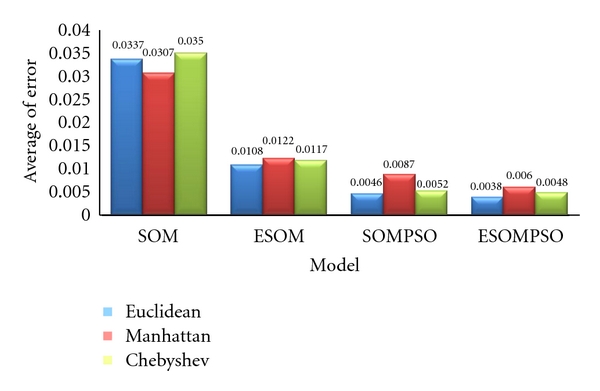
Quantization errors of SOM, ESOM, SOMPSO, and ESOMPSO.

Figures 4 and 5 depict the effectiveness of the ESOMPSO with better average accuracy and quantisation errors compared to the others. Regardless the types of distance measurements, the results of the proposed method are significant. This is due to the improved lattice structure and PSO in optimising the weights. As discussed before, the improved formulation of the hexagonal lattice structure gives more coverage on neighbourhood updating procedure. Hence, the probability for searching the salient nodes as winner nodes is higher, and this is presented in terms of accuracy and quantisation. However, the convergence time is slower for the proposed method due to the natural behaviour of the particles in searching for *g*best globally and locally. ESOMPSO with Euclidean distance gives the highest classification accuracy of 95.22% and the least quantisation error of 0.0038, accordingly.

However, this tradeoff, that is, higher accuracy with more convergence time and vice versa, does not give big impact on the success of the proposed methods due to the concept of *No Free Lunch Theorem *[42]. It means that general-purpose universal algorithm is impossible; an algorithm may be good at one class of problems, but its performance will suffer in the other problems. For detail explanation, higher accuracy is depending not only on types of datasets but also on the purpose of implementing the problems' undertaking. 

**Figure 6:**
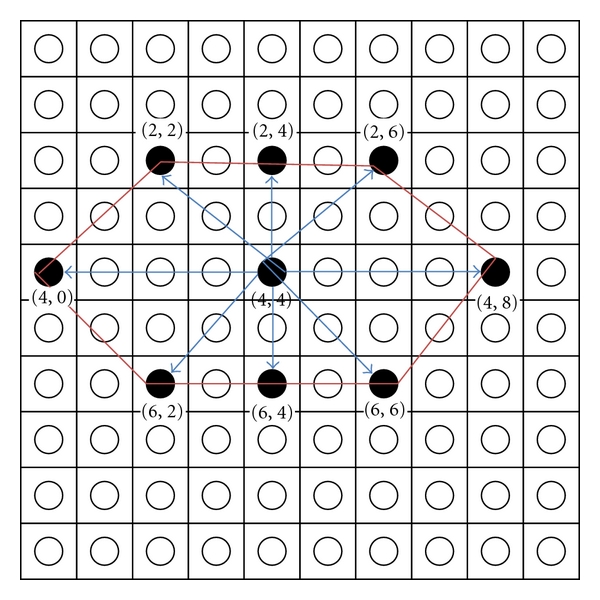
Improved hexagonal lattice structure.

From the findings, it seems that the selection of SOM's lattice structure for better learning is crucial in updating the neighbourhood structures for network learning. The standard formulation for basic and improved hexagonal lattice structure is illustrated in Figure 6. However, after training, the number of nodes to be updated was 10.39. Using the basic hexagonal formula, the wide area was not covered and caused insufficient neighborhood updating. The potential node might not be counted during the updating process. Now, we illustrate the scenario of the improved hexagonal lattice structure for wider and better coverage (Figure 6). Let say the BMU coordinate is (4, 4) with current radius, *r* = 2. The radius will decrease with exponential decay function. The improved neighborhood hexagonal lattice area is defined as (2).

By using improved hexagonal lattice area, the nodes will be updated to 33. The coverage area is better compared to the basic hexagonal lattice, and the potential nodes differences are 22.61. This formulation improves the neighborhood updating process; hence, better results of ESOMPSO are quite promising. The proposed methods are validated using the Kruskal-Wallis [43] test to probe the significance of the results. The experiments are implemented on all accuracy of the proposed methods, and the mean rank is generated as given in Table 6. It shows that the ESOMPSO with Euclidean distance generates higher mean rank. The table also illustrates that this method has yielded higher accuracy among others as claimed previously in our experiments. The generated *P* value is 0.004 which is less than the level of significant value of *α* = 0.05. Hence, the proposed methods have shown their dissimilarity among each other.

## 5. Conclusion

This paper presents multistrategy learning by proposing Enhanced Self-Organizing Map with Particle Swarm Optimization (ESOMPSO) for classification problems. The proposed method was successfully implemented on machine learning datasets: XOR, Cancer, Glass, Pendigits, and Iris. The analysis was done by comparing the results for each dataset produced by Self-Organising Map (SOM), Enhanced Self-Organising Map (ESOM), Self-Organizing Map with Particle Swarm Optimization (SOMPSO) and ESOMPSO with different distance measurements. The analysis reveals that ESOMPSO with Euclidean distance generate promising results based on the highest accuracy and the least quantization errors (referring to Figures 5 and 6) compared to SOM, ESOM, and SOMPSO for classification problems. This major impact of the proposed method is due to the improved formulation of the hexagonal lattice structure which gives more distributions and wider exploration and exploitation of the particle swarm optimization (PSO) particles to search for a better *g*best.

## Figures and Tables

**Algorithm 1:**
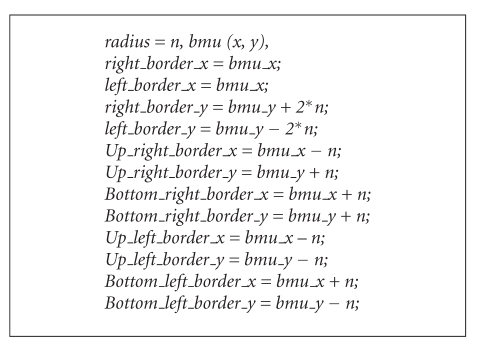
Algorithm for improved hexagonal lattice area.

**Table 1:** Data information.

Data type	Iris	XOR	Cancer	Glass	Pendigits
Input node	4	4	30	10	16
Output ode	1	1	1	1	1
Data size	150	8	569	214	10992
Training size	120	6	379	149	494
Testing size	30	2	190	65	498

**Table 2:** Class information.

Dataset	Number of class	Classes
Iris	3	Class 1: Iris Virginia
		Class 2: Iris Sentosa
		Class 3: Iris Versicolor
XOR	4	Class 1: 0
		Class 2: 1
Cancer	2	Class 1: Benign
		Class 2: Malignant
Glass	6	Class 1: Building windows float
		Class 2: Processed building
		Class 3: Windows float
		Class 4: Processed building
		Class 5: Windows nonfloat
		Class 6: Processed containers
		Class 7: Tableware Headlamps
Pendigits	10	Class 0: Digit 1
		Class 1: Digit 2
		Class 2: Digit 3
		Class 3: Digit 4
		Class 4: Digit 5
		Class 5: Digit 6
		Class 6: Digit 7
		Class 7: Digit 8
		Class 8: Digit 9

**Table 3:** Parameter settings for ESOMPSO.

Parameter	Dataset				
	Iris	XOR	Cancer	Glass	Pendigits
Input vector (Training)	120	6	379	149	7494
Input vector (Testing)	30	2	190	65	3498
Input dimension	4	4	30	9	16
SOM's Mapping Dimension (*X*, *Y*)	10 × 10	10 × 10	10 × 10	10 × 10	10 × 10
SOM lattice structure	Standard hexagonal	Standard hexagonal	Standard hexagonal	Standard hexagonal	Standard hexagonal
ESOM lattice structure	Improved Hexagonal	Improved hexagonal	Improved hexagonal	Improved hexagonal	Improved Hexagonal
Learning rate	0.5	0.5	0.5	0.5	0.5
Number of runs	10 times	10 times	10 times	10 times	10 times
Epoch	1000	1000	1000	1000	1000
*C*_1_	2.0	2.0	2.0	2.0	2.0
*C*_2_	2.0	2.0	2.0	2.0	2.0
Δ*t*	0.1	0.1	0.1	0.1	0.1
Number of particles	100	100	100	100	100
PSO problem dimension	10 × 10	10 × 10	10 × 10	10 × 10	10 × 10
Stop condition (minimum error)	0.0000193	0.0000193	0.0000193	0.0000193	0.0000193

**Table 4:** Summarization of SOM and ESOM results.

		SOM			ESOM		
		EUC	MAN	CHEBY	EUC	MAN	CHEBY
IRIS	Quantization error	**0.0348**	0.0358	0.0419	**0.0171**	0.0244	0.0275
	*Classification (%) *	**74.3333**	60.0000	70.0000	**76.6667**	73.3333	74.333
XOR	Quantization error	**0.2009**	0.2060	0.2159	**0.1941**	0.2458	0.2077
	*Classification (%) *	**75.3436**	68.4525	72.5632	**86.9876**	80.4462	84.2561
CANCER	Quantization error	**0.4541**	0.4913	0.5037	**0.4397**	0.4771	0.4781
	*Classification (%) *	37.8947	43.1579	**74.2105**	**77.8947**	34.7368	71.5789
GLASS	Quantization error	**0.0337**	0.0307	0.0350	**0.0108**	0.0122	0.0117
	*Classification (%) *	**50.9231**	13.8462	36.9231	**55.3846**	50.7692	44.6154
PENDIGITS	Quantization error	**0.1986**	0.2006	0.2103	**0.1897**	0.1957	1.2008
	*Classification (%) *	**74.6427**	44.5969	72.6415	**76.3579**	52.9445	69.1252

EUC: Euclidean distance, MAN: Manhattan distance, CHEBY: Chebyshev distance.

**Table 5:** Summarisation of SOMPSO and ESOMPSO results.

		SOMPSO			ESOMPSO		
		EUC	MAN	CHEBY	EUC	MAN	CHEBY
Iris	Epoch	1000	1000	1000	1000	1000	1000
	Quantisation error	**4.0799**	4.0979	4.0875	**1.8884**	2.0125	2.0565
	Convergence error	0.0318	0.0358	0.0322	0.0243	0.0587	0.0347
	Convergence time	22 sec	22 sec	22 sec	240 sec	240 sec	240 sec
	*Classification (%)*	**92.00**	89.24	90.45	**92.72**	90.11	90.75
XOR	Epoch	1000	1000	1000	1000	1000	1000
	Quantisation error	**0.5011**	0.6455	0.5866	**0.0048**	0.0250	0.0145
	Convergence error	0.2500	0.3204	0.3050	0.1916	0.2591	0.2641
	Convergence time	10 sec	10 sec	10 sec	17 sec	17 sec	17 sec
	*Classification (%)*	**94.11**	85.25	88.47	**95.22**	86.14	90.24
Cancer	Epoch	1000	1000	1000	1000	1000	1000
	Quantisation error	**0.0094**	0.0145	0.0102	**0.0050**	0.0125	0.0078
	Convergence error	0.5951	0.6523	0.6424	0.4422	0.5371	0.4823
	Convergence time	80 sec	80 sec	80 sec	110 sec	110 sec	110 sec
	*Classification (%)*	**90.69**	75.23	78.89	**91.77**	77.35	82.05
Glass	Epoch	1000	1000	1000	1000	1000	1000
	Quantisation error	**0.0046**	0.0087	0.0052	**0.0038**	0.0060	0.0048
	Convergence error	0.0435	0.0541	0.1242	0.0157	0.0324	0.0224
	Convergence time	40 sec	40 sec	40 sec	60 sec	60 sec	60 sec
	*Classification (%)*	**87.88**	80.98	84.66	**89.45**	82.45	84.87
Pendigits	Epoch	1000	1000	1000	1000	1000	1000
	Quantization error	**0.0458**	0.4752	0.4777	**0.0587**	0.5143	0.4221
	Convergence error	0.2060	0.2365	0.2241	0.1405	0.1569	0.1478
	Convergence time	110 sec	110 sec	110 sec	205 sec	205 sec	205 sec
	*Classification (%)*	**75.44**	70.25	72.48	**85.62**	70.85	72.89

EUC: Euclidean distance, MAN: Manhattan distance, CHEBY: Chebyshev distance.

**Table 6:** Kruskal-Willis ranks for the proposed methods.

Methods	Number of datasets	Mean rank based on accuracy (Euclidean distance)	Mean rank based on accuracy (the Manhattan distance)	Mean rank based on accuracy (the Chebyshev distance)
SOM	5	3.60	4.60	6.00
ESOM	5	8.40	7.60	7.00
SOMPSO	5	14.20	14.40	13.80
ESOMPSO	5	15.80	15.40	15.20

Total	20			

	*P*-value	0.004	0.008	0.025
